# The Cause and Cure of Disease in Horses’ Feet

**Published:** 1881-07

**Authors:** E. A. McLellan

**Affiliations:** Lecturer on Shoeing and Diseases of the Foot, Columbia Veterinary College and School of Comparative Medicine


### ART. XIII.—THE CAUSE AND CURE OF DISEASE IN HORSES' FEET.

EVER since the horse has been made the servant of man, efforts have been directed to the conservation of his feet, as upon their ability to withstand the wear to which they were subjected depended the usefulness of the animal. Before shoes were applied, the cultivation of a hardness in the horn, and a cupped shaped form of foot, were considered essential. Before the Christian era, Xenophen, the Athenian general, in some of his writings, gives particular directions in regard to the management of horses' feet. He says: "As attention must be paid to the horse's food, and exercise, that his body may be vigorous, so must care likewise be taken of his feet. Damp and smooth stable floors injure even naturally good hoofs. The floors should be made of irregularly shaped stones inserted in the ground, close to one another, similar to a horse's foot in size; for such stable floors give firmness to the feet of horses that stand on them." In treating of the duties pertaining to a commander of cavalry, he dwells on the necessity of attending to the horses' feet. "You must pay attention to their feet, so that they be in a condition to be ridden on rough ground, for when they suffer from being ridden they become useless." The trite saying of our own day, "No foot, no horse," is but the echo of these words of the ancient Greek historian. As the service required of the horse became greater, it became necessary to protect the horn from excessive wear, and shoes, or *solea*, as they were called, were constructed of various materials, such as straw, and skins of animals. In some cases horns were used for this purpose.

The date at which iron shoes were first applied to feet by nails is uncertain. Fleming, in his "Horseshoes and Horseshoeing," fixes it in the ninth century, and his researches in this field have been as exhaustive as those of any writer upon the subject.

According to the pattern of shoes shown in Fleming's work, in general appearance, though of ruder construction, they resembled our modern shoes, and fully answered the purpose intended, the protection of the hoof from excessive wear. From that time until the present, whenever horses have been subjected to the amount of labor for which their great strength and endurance adapted them, the iron shoe has furnished protection for the foot. In the newspapers of the day, we frequently read of attempts to use the horse without shoes, but such experiments are but going back to the custom which the ancients were forced to abandon. The wear, even with very moderate work upon our streets, would exceed the growth.

By the application of shoes, the hoofs were protected from excessive wear, and lameness from that cause prevented; but it soon became apparent that the application of shoes did not grant immunity from lameness, and to discover and remove the causes of lameness, and at the same time to properly and adequately protect the foot, has been the study and aim of horse owners, farriers and veterinary surgeons from that time to the present. Various have been the methods employed to accomplish these desirable results, but, comparing the literature upon the subject, ancient and modern, with my own observation, I am forced to believe that very little advancement has been made. In the sixteenth century, we have from the pen of an Italian the first treatise on shoeing, and in it are described forms of shoes which are in use to-day, and the same principles which Cæsar Fiaschi taught have been lauded in this century as new discoveries, which would revolutionize the whole system of shoeing. This treatise, written in 1564, forms the basis of nearly all the literature connected with horseshoeing. The various writers upon the subject since that time have advocated different methods of preparing the hoof for the shoe, and other details connected with the business, some believing that a free use of the knife was necessary, and others that very little paring was needed; writers differ also in regard to the shoe, some believing that lightness is desirable, others that a heavy broad shoe is necessary for protection; some that the bearing of the shoe should be limited to the wall, while others advocated its resting also upon the sole; some, that the frog should be made a weight bearer, and others that it was not designed for that purpose. Whatever theory was advanced, it was usually made applicable to all cases.

Contraction of the hoof has always held a prominent place in the causation of lameness, and scores of shoes designed to relieve this condition have been patented. In addition, various medicinal preparations for the prevention and removal of lameness have, from time to time, appeared, and yet our horses suffer from lameness. Lameness arising directly or indirectly from abnormal conditions of the feet, is the prominent symptom in the majority of cases which are brought to the veterinary surgeon for treatment. There seems to have been no mitigation of the evil, but rather an aggravation of it in modern times. This condition of affairs has suggested to the writer these queries:

*First.—Have the true causes of lameness been made prominent by writers upon the subject?*

*Second.—How shall the horse's foot be perfected and preserved?*

In reference to the first query, I feel justified, after an extensive acquaintance with the literature upon the subject, coupled with the experimental knowledge gained by many years' experience in the shoeing and management of horses' feet, in answering this question in the negative.

I shall not attempt to point out all the causes of lameness, but shall endeavor to make clear what I consider to be the most frequent cause.

To do this it will be necessary to refer to the construction of the foot. The foot of the horse is formed by the enclosure of the last joint of the extremity in horn, and it must be evident that a certain height of horn is required upon each side, and at the toe and heel, to place the joint enclosed in its right relation to the bone immediately above it.

This horn is attached by means of the laminæ, which ensure a strong and elastic union. At the superior internal border is the coronary concavity, which lodges the horn-producing tissues, and changes in the bearing of the foot interfere with the function of this part, modifying the secretion of horn. Through its laminal attachment, the bone is suspended within the hoof, and is surrounded by about three-eighths of an inch of highly organized and sensitive tissue. That these tissues be not injured either by pressure or tension, it is necessary that the right relation of the different parts be maintained, and it is the disturbance of this relation which is the primary cause of the diseased conditions of the foot. Thus, when the hoof becomes so changed in form, or the position of the pedal bone altered in its relation to the hoof, so as to bruise the tissue lying between the wings of the bone and hoof, it produces that redness at the heels popularly known as corns. This redness in the plantar horn is frequently seen at other parts of the sole, but wherever seen is produced in a similar manner. Excessive and unequal pressure at the coronary concavity is a very common condition to be found. The inside border is more likely to suffer than the outer from this cause, and it always results in a modified secretion of horn, the hoof becoming thin and subject to fissures. In a similar manner, diseases of the lateral cartilage arise, and a tendency to lammitis and navicular disease is developed.

I have in my possession a foot and leg of a horse, which presents some changes in the relation of hoof and parts enclosed, with some of the effects of the same. To prepare the specimen I removed the skin from the leg as far up as the knee, and hung it up to dry. About a year afterward I examined it, and found the soft tissues surrounding the pedal bone all removed; although externally the foot presented the same appearance as when it was hung up. I removed sections of the horn from superior to inferior borders, large enough to permit of examination without disturbing the relation of the parts. The hoof was turned to one side by unequal growth, but the bones had endeavored to preserve the straight line, and, as a consequence, the wall was quite close to the bone upon one side, and quite removed from it on the other. Upon one border of the plantar surface the bone rested upon the sole, while from the other it was unduly elevated. Where the bone rested upon the sole, the sole was extremely thin. At the coronary border the pressure was greatest at the inner quarter, and as a consequence the wall here was thinnest.

We can imagine the suffering which must have accompanied these changes. This case, although an aggravated one, is not exceptional. The horse was young, had been purchased as a sound horse less than a year before, being put to work on a cart for which he was suited. At first an awkward gait was noticeable—an uneven wearing of the shoes—later, tenderness in the feet, which eventually increased to such extent as to be recognized as lameness. This lameness was not excessive at any time, and would have been accounted for ordinarily by the owner in various ways, such as *general* abuse by the driver, excessive feeding, watering when heated, driving shoes on too tightly, hot fitting, applying a shoe too small to protect the foot, etc., the true cause of the lameness being entirely overlooked. I mention this case as illustrative of what I conceive to be the most prominent cause of lameness, *viz.*, *a lack of harmony in the relation of the different parts comprising the foot.* All feet are more or less affected in this manner. The condition seems almost inseparably connected with the artificial surroundings of the horse, but if generally recognized as the *source* of lameness, efforts will be directed towards mitigating the evil. All horses, it is true, are not lame. Some are less sensitive than others; but a better reason for such exemption is that some feet, from their form, are less susceptible to injury than others, from changes in the foot. These changes, although they may not be sufficient to produce that irregular motion recognized as lameness, yet they do interfere with that regular, steady, machine-like action which the horse, perfectly balanced upon his feet, will exhibit. Interfering, overreaching, excessive wear of the shoe at the toe, or upon one side, or at the heels, standing with the feet too far forward or backward, are all indications of a lack of balance or harmony between the parts. If these are facts, they are such as every horseshoer and veterinarian should be familiar with. They should be able to know from an examination of the foot just the condition of the parts within, and the best course to adopt to preserve the foot if perfect, and restore to health if diseased.

It will be admitted that this knowledge is not possessed by the majority of horseshoers.

With many the perfection of the art consists in paring out the sole smoothly, the selection of a shoe which suits the fancy of the owner, modifying it to fit exactly the outline of the foot, driving the nails evenly, and finishing off with most artistic filing of the hoof, with no thought whatever as to whether there is equal pressure at the coronet, or to the position of the joints which make up the limb.

In securing these ends, it will frequently be found necessary to change the form of the shoe from the ordinary shape, in some instances making the heels thick, and in others thin. And the same holds good in regard to the side, one may be thicker than the other. These rules are applicable not alone to the old and diseased, but to all ages. Colts that are shod for the first time often exhibits as great defects as any.

To recapitulate: *the most frequent cause of lameness is a disturbance of the relations of the parts within the hoof, and to preserve the foot it is necessary to secure and maintain all parts of the foot in their normal relations.* This is the fundamental truth which should direct all efforts for the preservation of the foot. If this is not secured, friction and disease are sure to follow.

If these principles are true, and so essential, it follows that any treatise purporting to teach the business of horseshoeing should seek to enforce these primary facts, and following this, that the different forms of feet should be illustrated, and the abnormal conditions within the hoofs most likely to be developed from the peculiar form of foot presented, together with the best method of defeating that tendency and preserving the foot, be fully explained. If the books which have been written to teach that the whole art of horseshoeing is embraced in preserving the frog from the knife, and allowing it to reach the ground, had enforced these truths, or, if the effort expended in illustrating the idea that all lameness was due to a too free use of rasp and knife, had been expended in behalf of these fundamental facts, horseshoeing might now be what it is not, the conservation of the foot, rather than its destruction.

*Second.—How shall the horse's foot be perfected and preserved?*

If we are ever to have perfect feet, we must begin and develop them. A recent writer has said, "No two peculiarities of the horse are more unerringly transmitted than the eyes and feet," a fact confirmed in my own experience in numberless instances. This is a fact which every man should keep in mind in selecting animals to breed from.

Opinions differ as to what is a desirable form of foot. My own is that the mule-shaped foot is the best, the one least likely to change its form with age and shoeing. One point that I consider most essential is, that the heels should be long posteriorly, that this elastic portion of the foot should be well developed. The short-heeled foot (by shortness I do not refer to the height of horn, but to its length posteriorly), wider across the quarter than it is long, is particularly undesirable. This form is liable to change, and these changes are more quickly followed by lameness than in the long-heeled form. This difference between the long and short heel can be explained in this manner: There is a tendency in the hoof shod to develop anteriorly, and to become less and less posteriorly. This carries the bearing surface of the foot further forward, and places it at a disadvantage in supporting the weight of the animal. A foot that is short in the heels is already too much developed at the toe. Again, the hoof is likely to become narrow in the heels, from a variety of causes which we will not stop here to enumerate. If the heels are long, this condition scarcely affects the gait of the horse, but if short, the contraction will be hardly perceptible before lameness will be produced.

As soon as the colt is born, the care of its feet should commence. Almost all writers upon the subject seem to agree that the surroundings of the colt have very much to do with the shape of the feet, *i. e.*, if i tis raised upon dry ground its feet incline to be narrow and hollow, and if on wet soil to be wide and flat. These forms are modified by inheritance. Moist conditions are undesirable, as producing the width and consequent shortness of the heels.

Whatever shape is considered desirable should be cultivated by intelligent use of knife and rasp, or even by the application of shoes, where there is a tendency to spread too much at the plantar surface. During the winter season, when the growth exceeds the wear, they require frequent use of rasp. In summer the wear is sometimes uneven, and this must be regulated.

If, with this object to be attained, a judicious selection of sire and dam has been made, if the circumstances attending the growth of the colt have been favorable, as a result we shall have a perfect foot, which, if not abused, will last as long as any other part of the animal, that is to say, from twenty to thirty years.

Feet, then, are to be perfected by the cultivation of a perfect form, and they are to be preserved in shoeing by such intelligent use of knife and application of shoes as shall maintain the right relation between all the parts of the foot and limb, thus securing the proper physiological function of each part, and giving to the horse that bold, even, graceful stride which is pleasure to the animal and the pride of his owner.

This article has already exceeded the limit intended, and we leave for some future occasion further reference to the care of the feet in the stable in sickness and health.

E. A. McLellan,
*Lecturer on Shoeing and Diseases of the Foot,*
*Columbia Veterinary College and*
*School of Comparative Medicine.*

